# Acupuncture for Mild Cognitive Impairment and Dementia: An Overview of Systematic Reviews

**DOI:** 10.3389/fnagi.2021.647629

**Published:** 2021-05-14

**Authors:** Wenbo He, Meixuan Li, Xuemei Han, Wei Zhang

**Affiliations:** ^1^Institute of Hospital Management, West China Hospital, Sichuan University, Chengdu, China; ^2^Evidence Based Medicine Center, School of Basic Medical Sciences, Lanzhou University, Lanzhou, China; ^3^School of Public Health, Lanzhou University, Lanzhou, China; ^4^West China Biomedical Big Data Center, West China Hospital, Sichuan University, Chengdu, China

**Keywords:** acupuncture, mild cognitive impairment, dementia, overview, systematic review

## Abstract

**Background:** Dementia is a gradual decline in cognitive ability and is becoming more common in our elderly population. Mild cognitive impairment (MCI) is defined as a slight clinical deterioration of memory capacity, below the level of normal aging, but does not constitute a clinical diagnosis of dementia. To date, no interventions have been proven to cure MCI and dementia fully.

**Purpose:** To evaluate the potential effectiveness and safety of acupuncture for mild cognitive impairment (MCI) and dementia and evaluate the methodological quality of systematic reviews (SRs).

**Methods:** We conducted a literature search for SRs with meta-analyses in seven Chinese and international databases through October 1, 2020. The basic characteristics of the included SRs/meta-analyses and the basic information of the original included randomized controlled trials were extracted by three reviewers independently. A meta-analysis of the original randomized controlled trials from the included SRs/meta-analyses was performed using Stata 12.0 software. The Assessing the Methodological Quality of Systematic Reviews 2 was used to assess the methodological quality of the included SRs/meta-analyses, and the Grading of Recommendations, Assessment, Development, and Evaluation was used to rate the quality of evidence.

**Results:** A total of 35 SRs/meta-analyses were included, and the majority showed that acupuncture was more effective than western medicine or conventional therapy for MCI and dementia [odds ratio =1.39; 95% confidence interval (CI): 1.24, 1.56]. There was a statistically significant difference in the Mini-Mental State Examination score (weighted mean difference = 1.23; 95% CI: 0.78, 1.68; *p* < 0.00001), and there was no significant improvement in the activities of daily living score (weighted mean difference = 1.58; 95% CI: −0.02, 3.18; *p* = 0.053). The assessment results of Assessing the Methodological Quality of Systematic Reviews 2 showed that the methodological quality of most included SRs/meta-analyses was critically low; the lowest scores were items 2, 7, and 10. For Grading of Recommendations, Assessment, Development, and Evaluation, of the 73 outcomes, 50 (68.5%) outcomes were low or very low quality, and 23 (31.5%) outcomes were moderate quality.

**Conclusions:** Acupuncture can be considered as an alternative for the treatment of MCI and dementia when western medicine or other therapies are contraindicated. More high-quality evidence is needed to determine further the effectiveness of acupuncture.

## Introduction

Dementia is characterized by progressive cognitive fail and is becoming more rifeness in an older person (Cunningham et al., 2015). With an aging worldwide population, the prevalence of dementia is increasing every year. Approximately 50% of people older than 80 years have dementia, and up to 4.6 million new cases are diagnosed per year (Kosenko et al., 2017). Dementia has become an important cause of premature death (Prince et al., 2013).

Mild cognitive impairment (MCI) is defined as a slight clinical deterioration of memory capacity, below the level of normal aging, but does not constitute a clinical diagnosis of dementia (Gauthier et al., 2006); it is the interim phase between regular cognitive functioning and dementia (Karssemeijer et al., 2017). Patients with MCI have a high probability of developing dementia but have not yet reached an irreversible stage (Kim et al., 2019). Approximately one-fifth of cases of MCI will evolve into dementia every year (Gauthier et al., 2006). Dementia and MCI seriously influence the patients' health and life quality and place a heavy burden on families and society (Wang et al., 2019).

Acupuncture is a treatment modality that involves the insertion of needles into the body (Cao et al., 2014). The therapeutic mechanism of acupuncture may involve regulation of the release of central neurotransmitters, the resistance of neurons to oxidative damage and removal of free radicals, inhibition of neuronal apoptosis, inhibition of inflammatory reactions in brain tissue, activation of hippocampal protein kinases, and inhibition of the expression of tau protein related to microvessels (Tian et al., 2012). Some clinical studies have found that acupuncture can significantly reduce serum total cholesterol in patients and exert a positive effect on cerebral hemodynamics (Hou and Bu, 2000; Shang and Ding, 2003). At the same time, acupuncture is simple and cheap and can avoid the toxicities and adverse effects of drugs and reduce medical investment. However, further research into its mechanisms of action is necessary to take full advantage of the potential benefits of acupuncture for MCI and dementia.

To date, no interventions have been found to cure MCI or dementia fully. However, some studies suggest that acupuncture might be beneficial in these conditions. Acupuncture has been used empirically for thousands of years (Kim et al., 2019). Previous research has stated that acupuncture may be an effective adjuvant treatment for neurological diseases, such as MCI (Mai and Zheng, 2015), post-stroke cognitive impairment (Yuan and Zhang, 2010), vascular dementia (Wu et al., 2018), and Alzheimer's disease (Kosenko et al., 2017).

This review aimed to comprehensively evaluate the published study on the use of acupuncture for MCI and dementia to clarify further its safety and effectiveness. We performed a systematic review (SR) to reanalyze all available SRs and meta-analyses, access the methodological quality of these SRs/meta-analyses, and evaluate the evidence quality.

## Materials and Methods

### Search Strategy

This SR of SRs was carried out based on the Preferred Reporting Items for Systematic Reviews and Meta-Analyses statement and the Cochrane handbook (Gerard and Xavier, 2010; Green, 2011). PubMed, Web of Science, EMBASE, China National Knowledge Infrastructure, Cochrane Library, VIP Periodical Resource Integration Service Platform, and Chinese Biomedical Databases were searched up to October 1, 2020.

Search terms were as follows: (“acupuncture therapy” OR “acupuncture” OR “acupuncture ear” OR “electro-acupuncture” OR “auricular acupuncture” OR “auricular needle” OR “scalp acupuncture” OR “catgut implantation” OR “acupuncture points” OR “abdominal acupuncture”) AND (“Amentia” OR “Alzheimer's Disease” OR “ATD” OR “Alzheimer Type Senile Dementia” OR “Alzheimer Disease” OR “Alzheimer Type Dementia” OR “Alzheimer Syndrome” OR “Alzheimer Dementia” OR “Cognitive Dysfunction” OR “Senile Dementia” OR “Primary Senile Degenerative Dementia” OR “MCI” OR “Mild Neurocognitive Disorder” OR “Cognitive Impairment” OR “Mild Cognitive Impairment” OR “Cognitive Decline” OR “Mental Deterioration” OR “Age-Related Memory Disorders” OR “Dementia” OR “Senile Paranoid Dementia”) AND (“Systematic” OR “Review” OR “Meta-analysis”) (Appendix A in Supplementary Material). We also manually searched any included articles for additional relevant SRs/meta-analyses.

### Inclusion Criteria

1. Study design: SRs/meta-analyses of randomized controlled trials (RCTs) of acupuncture for patients with MCI or dementia.
2. Subjects: patients who have been diagnosed with dementia or MCI, and there is no restriction on sex, age, and race.
3. Treatment group intervention: acupuncture (manual acupuncture or electroacupuncture (EA) of the body, scalp, or ear) alone or acupuncture added other measures.
4. Control group intervention: Western medicine (including Nimodipine, Huperzine, Perphenazine, Duxil, Donepezil, and Hydergine), cognitive intervention, routine treatment, no intervention, etc.
5. Language: Chinese or English.

### Exclusion Criteria

1. Treatment group did not receive acupuncture or used it only as a supplementary therapy
2. Provision of acupuncture as an intervention in the control group
3. Studies providing insufficient information for evaluation
4. Duplicate publications, commentaries, or meeting abstracts.

### Literature Screening and Data Extraction

All retrieved studies were imported into EndNote X7 software. This study excluded documents with repeated publications and data reuse, and three authors independently screened titles and abstracts to identify eligible studies. Full texts were extracted, and articles that met the inclusion criteria were read to determine whether they would enter the final analysis. The authors determined any conflicts through discussion or negotiation with the fourth member of the review team.

Data were extracted from the included SRs/meta-analyses. Based on the characteristics of the included studies, we extracted the following basic information from each included review: first author, publication year, country, quality assessment tools, number of included articles, sample size, treatment and control interventions, and outcomes.

Considering the inconsistency of the results of the included SRs/meta-analyses, we screened the original RCTs and conducted a new meta-analysis. The main information extracted for this purpose was as follows: first author, publication year, number of participants, intervention and control measures, disease types, and outcome measures.

### Quality Assessment

Two reviewers independently assessed the quality of included SRs/meta-analyses using the A Measurement Tool to Assess Systematic Reviews 2 (AMSTAR-2) checklist, which includes 16 items, of which seven are considered key domains (Shea et al., 2017). In addition, AMSTAR-2 divides the overall confidence in the review results into one of four levels: high, moderate, low, or critically low. The assessment options are limited to the following three: “Yes,” “Partial Yes,” and “No.” To generate a numerical score, we assigned 1 for “Yes,” 0 for “No,” and 0.5 for “Partial Yes” in this SR. We used the Cochrane Collaboration tool to evaluate the risk of bias of the included original RCTs.

### Data Synthesis

The Cochrane Handbook was used to assess the effectiveness of acupuncture at the SR level (Green, 2011). Meta-analysis was implemented using Stata 12.0 (StataCorp, College Station, TX, USA). Odds ratios (ORs) and 95% confidence intervals (CIs) were used to estimate clinical efficacy/effectiveness, while continuous data was showed as weighted mean differences (WMDs) and 95% CIs. I^2^ values were used to assess the heterogeneity among RCTs (I^2^ > 50% showed the existence of heterogeneity).

## Results

### Study Identification

**Figure 1:**
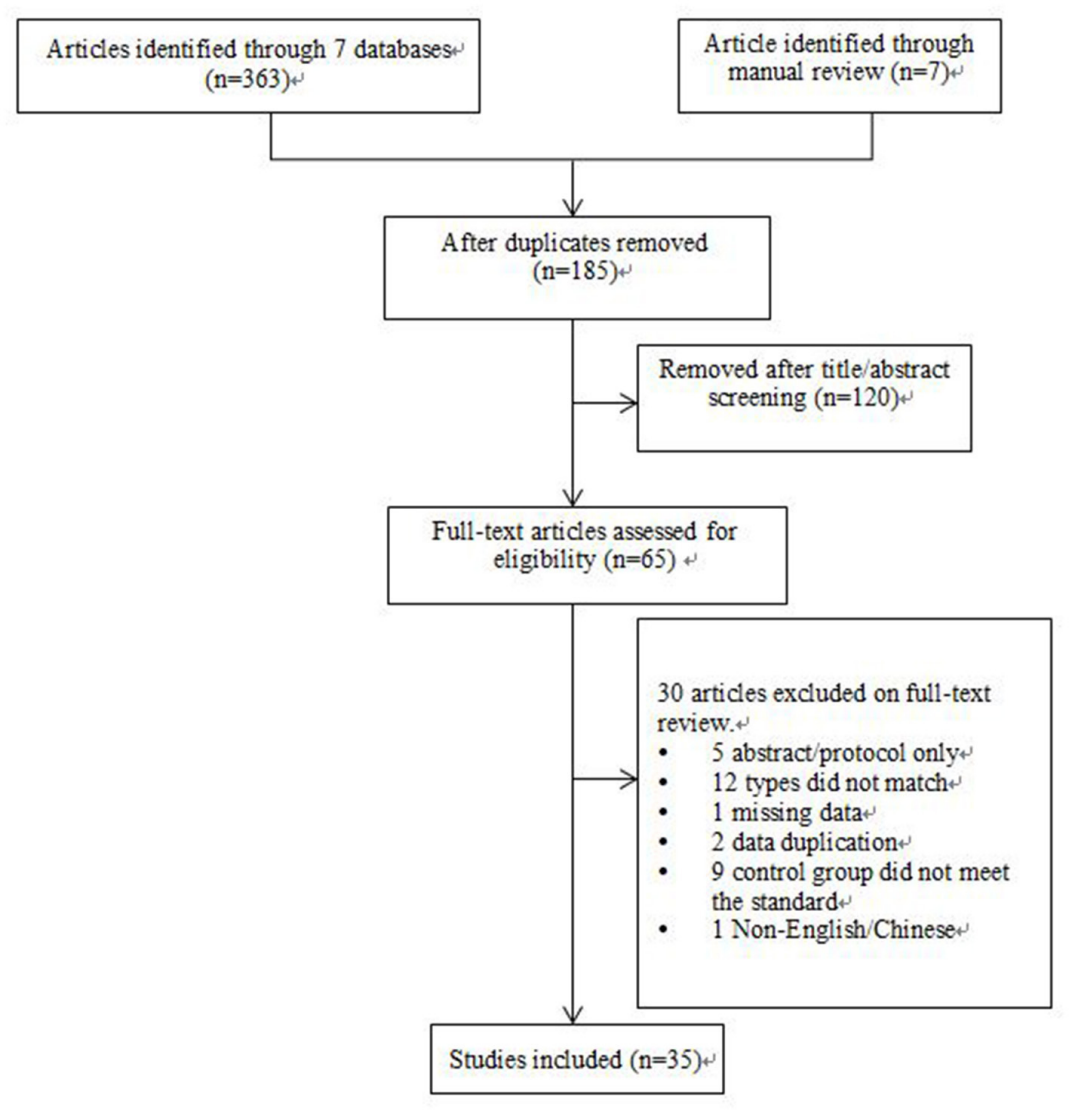
Flow diagram of article selection for inclusion according to the PRISMA guidelines.

Three hundred seventy reviews were retrieved from the database searches, of which 65 remained after elimination of duplicates. Of these, 30 articles were excluded based on title, abstract, and full-text screening (Appendix B in Supplementary Material), and 35 studies met all the criteria of inclusion and were included in this overview. Details on the study screening process are shown in Figure 1.

### Characteristics of Included Reviews

The 35 SRs/meta-analyses were published between 2007 and 2020. Thirty-two SRs/meta-analyses were conducted in China (Peng et al., 2007; Guo et al., 2008; Zhu and Zhang, 2009; Yuan and Zhang, 2010; Liu et al., 2011, 2015, 2018; Tian et al., 2012; Cao et al., 2013, 2014; Fang et al., 2014; Hu et al., 2014; Zhao et al., 2014; Lu et al., 2015; Mai and Zheng, 2015; Xu and Xie, 2015; Zhang et al., 2015; Zhou et al., 2015, 2017; Deng and Wang, 2016; Lin et al., 2016, 2017; Xiong et al., 2016; Zou et al., 2016; Wan et al., 2017; Zhan et al., 2017; Li et al., 2018, 2020; Wu et al., 2018; Tang et al., 2020; Wang et al., 2020), and three SRs/meta-analyses were conducted in Korea (Lee et al., 2008; Kwon et al., 2018; Kim et al., 2019). Twenty-three SRs/meta-analyses (68%) were published in Chinese, and 12 (34%) were published in English. Characteristics of these SRs/meta-analyses can be found in Table 1.

**Table 1:** Characteristics of included systematic reviews and meta-analyses of randomized controlled trials.

**References**	**Country**	**Quality assessment tools**	**Number of RCTs (number of participants)**	**Type of disease**	**Intervention**	**Comparator**	**Outcomes**	**Meta-analysis**
Peng et al. (2007)	English	Author defined	—	Vascular dementia	—	—	—	N
Guo et al. (2008)	Chinese	Jadad	22 (1,378)	Alzheimer	EA	Nimodipine	General effectiveness, MMSE	Y
Lee et al. (2008)	English	Jadad	3 (166)	Alzheimer	EA	Nimodipine, Huperzine, Perphenazine	MMSE, ADLs	Y
Zhu and Zhang (2009)	Chinese	Jadad	7 (517)	Vascular dementia	EA	Nimodipine,Duxil	General effectiveness, MMSE	Y
Yuan and Zhang (2010)	Chinese	Jadad	9 (615)	Post-stroke CI	Acupuncture + Nimodipine	CFT	MMSE	Y
Liu et al. (2011)	Chinese	Jadad	6 (330)	MCI	Acupuncture + Nimodipine/Donepezil/ Duxil	Nimodipine, Donepezil, Duxil	General effectiveness, MMSE	Y
Tian et al. (2012)	Chinese	Cochrane Handbook	8 (339)	Alzheimer	Acupuncture	Nimodipine, Donepezil, Duxil	MMSE	N
Cao et al. (2013)	English	Cochrane Handbook	12 (691)	Vascular MCI	BA + CFT	Nimodipine, Donepezil, CFT	MMSE	Y
Fang et al. (2014)	English	Cochrane Handbook	21 (1,421)	Post-stroke CI	Acupuncture + CFT	CFT	MMSE	Y
Zhao et al. (2014)	Chinese	Jadad	16 (1,103)	Post-stroke CI	EA/Acupuncture/SA + CFT/Nimodipine	Nimodipine, CFT	General effectiveness, MMSE, ADL	Y
Cao et al. (2014)	Chinese	Cochrane Handbook	5 (233)	Alzheimer	Acupuncture	Nimodipine, Donepezil, Duxil	MMSE, ADLs	Y
Hu et al. (2014)	Chinese	Cochrane Handbook	(1,092)	MCI	Acupuncture + Nimodipine	Nimodipine, CFT	General effectiveness, MMSE	Y
Zhou et al. (2015)	English	Cochrane Handbook	10 (585)	Alzheimer	Acupuncture, EA	Nimodipine	MMSE, ADLs	Y
Liu et al. (2015)	Chinese	Jadad	8 (578)	Post-stroke CI	EA	CFT	General effectiveness, MMSE	N
Mai and Zheng (2015)	Chinese	Jadad	5 (565)	MCI	EA	CFT	General effectiveness, MMSE, MoCA	Y
Lu et al. (2015)	Chinese	Cochrane Handbook	8 (720)	Ischemic type CI	Acupuncture + CT	CT	General effectiveness, MMSE, ADLs	Y
Zhang et al. (2015)	Chinese	Cochrane Handbook	11 (789)	Post-stroke CI	Acupuncture + CFT	CFT	General effectiveness, MMSE, ADLs	Y
Xu and Xie (2015)	Chinese	Jadad	10 (652)	Alzheimer	BA + Nimodipine	Nimodipine	General effectiveness, MMSE, ADLs	Y
Deng and Wang (2016)	English	Cochrane Handbook	5 (568)	Amnestic MCI	SEA	Nimodipine	General effectiveness, MMSE	Y
Xiong et al. (2016)	Chinese	Cochrane Handbook	13 (1,113)	Post-stroke CI	SA + CT	CT	General effectiveness, MMSE	Y
Lin et al. (2016)	Chinese	NR	19 (1,275)	Post-stroke CI	Acupuncture	CT	MMSE, ADLs, MoCA	Y
Zou et al. (2016)	Chinese	Cochrane Handbook	8 (349)	Alzheimer	Acupuncture	Huperzine, Nimodipine	General effectiveness, MMSE, ADLs, HDS	Y
Zhou et al. (2017)	English	Cochrane Handbook	15 (1,217)	Alzheimer	Acupuncture + HM	Huperzine, Hydergine, Donepezil	General effectiveness, MMSE, ADLs	Y
Wan et al. (2017)	Chinese	Jadad	6 (435)	Vascular dementia	EA	Nimodipine	General effectiveness, MMSE, ADLs	Y
Zhan et al. (2017)	Chinese	Cochrane Handbook	14 (896)	Post-stroke CI	EA + Nimodipine/CFT	Nimodipine/CFT	General effectiveness, MMSE, MoCA	Y
Lin et al. (2017)	Chinese	Cochrane Handbook	13 (751)	Alzheimer	Acupuncture	TCM	General effectiveness, MMSE	Y
Kwon et al. (2018)	English	Cochrane Handbook	9 (677)	CI	AA	HM/Nimodipine, Almitrine-Raubasine	General effectiveness, MMSE, ADLs	Y
Wu et al. (2018)	Chinese	Jadad	9 (656)	Vascular dementia	EA	Nimodipine	General effectiveness, MMSE, ADLs	Y
Liu et al. (2018)	Chinese	Cochrane Handbook	22 (1,637)	Post-stroke CI	EA + CT + CFT	CT + CFT	MMSE	Y
Li et al. (2018)	Chinese	Cochrane Handbook	10 (666)	MCI	Acupuncture + Nimodipine	Nimodipine	General effectiveness, MMSE, ADLs, MoCA	Y
Kim et al. (2019)	English	Cochrane Handbook	5 (257)	MCI	EA	Nimodipine, Donepezil	MMSE, MoCA	Y
Wang et al. (2019)	Chinese	Cochrane Handbook	8 (472)	Alzheimer	Acupuncture + Donepezil/ Huperzine	Donepezil, Huperzine	MMSE, ADLs	Y
Li et al. (2020)	English	Cochrane Handbook	15 (1,049)	MCI	Acupuncture + Nimodipine	Nimodipine/ Donepezil/CFT	General effective, MMSE, MoCA, ADLs	Y
Wang et al. (2020)	English	Cochrane Handbook	25 (2,035)	Alzheimer	Acupuncture/EA	Nimodipine/ Donepezil/Huperzine	MMSE, ADLs, HDS	Y
Tang et al. (2020)	English	Cochrane Handbook	16 (1,241)	Post-operative CI	Acupuncture	CFT	Incidence of post-operative CI, MMSE	Y

*NR, not reported; RCT, randomized controlled trial; CI, cognitive impairment; MCI, mild cognitive impairment; EA, electroacupuncture; CFT, cognitive function training; AA, auricular acupuncture; HM, herbal medicine; SA, scalp acupuncture; SEA, scalp electroacupuncture; CT, conventional treatment; BA, body acupuncture; HDS, Hasegawa Dementia Scale; MoCA, Montreal Cognitive Assessment; MMSE, Mini-Mental State Examination; TCM, Traditional Chinese Medicine; ADL, activity of daily living; Y, Yes; N, No*.

The 52 original RCTs contained in these 35 SRs were published between 1997 and 2018. We extracted information such as the length of the acupuncture process and traditional acupuncture points used, which mainly included GV20 (Baihui), Sishencong, LR3 (Taichong), and ST36 (Zusanli). The participants in the original study were all from hospitals, and the acupuncture interventions were performed by professional medical staff. Characteristics of the original RCTs can be found in Appendix C in Supplementary Material.

### Risk of Bias in Included Original Trials

**Figure 2:**
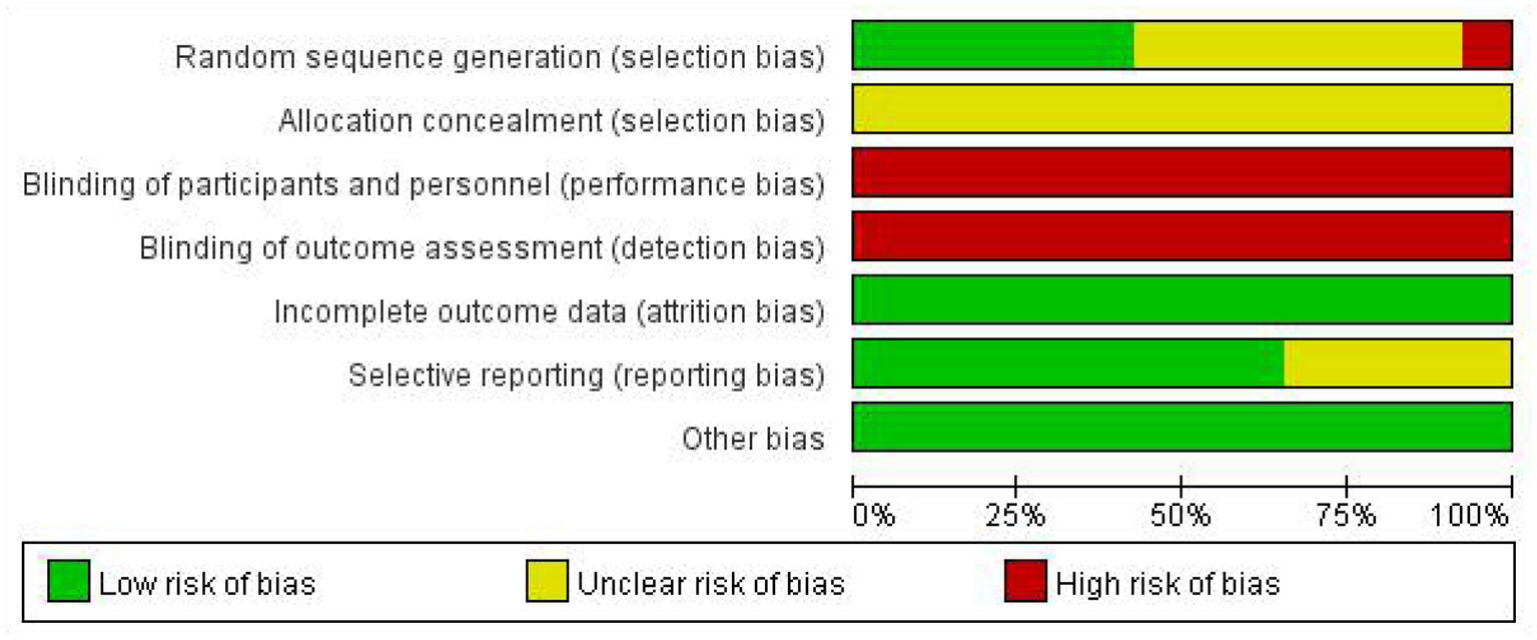
Risk of bias graph.

**Figure 3:**
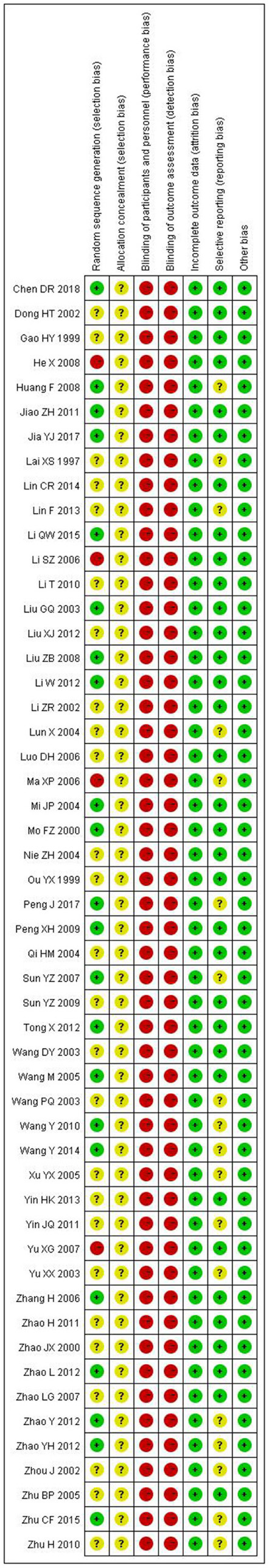
Risk of bias summary.

According to the seven risks of bias domains, the authors conducted a graphical summary of bias risk assessment for the 52 included studies (see Figures 2, 3). Of these 52 studies, patients were randomized using SPSS software or a random number table in 22. The remaining 30 studies referred to “randomization” but did not describe specific randomization methods. None of the literature described allocation concealment. Due to the particularity of acupuncture, blinding was not attempted in any articles. All the included literature had complete data for analysis. The outcome indicators of 18 articles were not comprehensive enough, but the analysis results of this study were not affected.

### Evaluation of Therapeutic Effect

#### Effectiveness Rate

**Figure 4:**
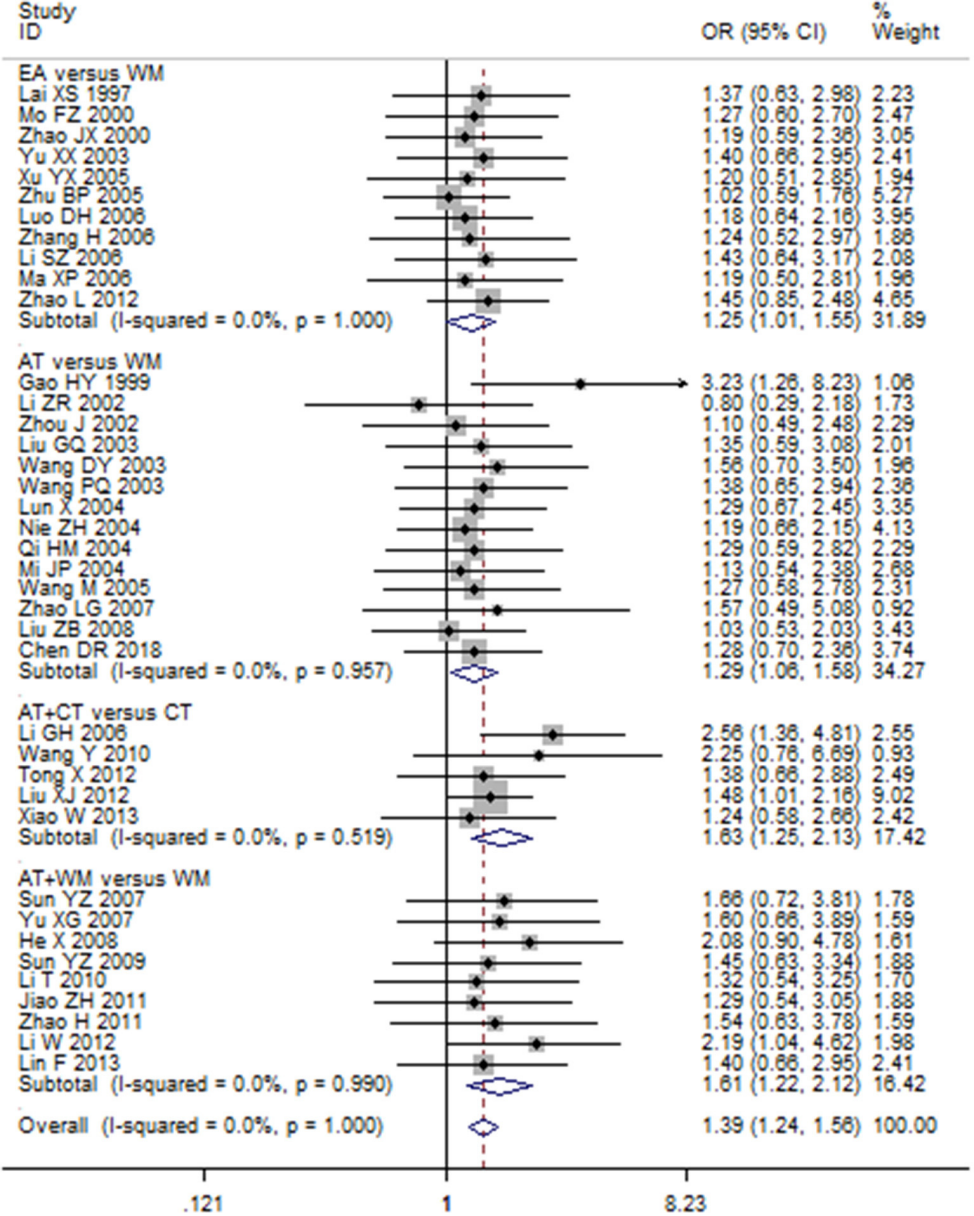
Forest plot of the effect of acupuncture on the clinical efficacy using fixed model.

Totally, 18 combined results were reporting on the effectiveness rate of acupuncture for MCI and dementia (Appendix D in Supplementary Material), and the results showed that acupuncture (including EA, scalp acupuncture, scalp EA, and body acupuncture) was generally superior to control interventions (Nimodipine, Huperzine, Perphenazine, Duxil, Donepezil, Hydergine, sham acupuncture, and cognitive function training) in improving MCI or dementia. Our new meta-analysis demonstrated an increased effectiveness rate in acupuncture vs. control groups across all RCTs using a fixed-effects model (OR 1.39, 95% CI 1.24–1.56; *p* < 0.001) (Figure 4).

#### Mini-Mental State Examination Score

The MMSE scale was used to assess the overall changes in cognition before and after treatment. Thirty-one pooled results described cognitive function measured by MMSE (Appendix D in Supplementary Material). Among them, 26 pooled results showed higher scores in the intervention group than the control group. However, five pooled results (Lee et al., 2008; Cao et al., 2014; Zou et al., 2016; Lin et al., 2017; Kwon et al., 2018) showed no significant difference between them. We extracted the original RCTs from the included SRs/meta-analyses and processed a reanalysis, dividing them into subgroups based on the two major disease categories (MCI and dementia). In the subgroup of dementia vs. Western medicine, the WMD was 1.26 (95% CI 0.58–1.94; *p* < 0.001) using the random-effects model; in the subgroup of MCI vs. Western medicine, the WMD was 1.09 (95% CI 0.72–1.46; *p* < 0.001) using a random-effects model. There was a statistically significant difference between acupuncture and control groups (Appendix F in Supplementary Material).

#### Self-Care Ability

Activity of daily living (ADL) scores were used to assess self-care ability after the intervention. A total of 16 pooled results showed the effects of acupuncture on ADLs in patients with cognitive impairment or dementia (Appendix D in Supplementary Material). Eight pooled results showed that acupuncture was more effective than the control interventions, but six results (Cao et al., 2014; Zou et al., 2016; Wan et al., 2017; Zhou et al., 2017; Kwon et al., 2018; Wu et al., 2018) found no difference between the two groups. Through the reanalysis of the original RCTs, the WMD was 1.58 (95% CI −0.02 to 3.18; *p* = 0.053) using the random-effects model, with no statistically significant difference between acupuncture and control groups (Appendix G in Supplementary Material).

### Publication Bias and Sensitivity Analysis

We used funnel plots to evaluate publication bias of the extracted original RCTs and were not completely symmetrical, indicating that there might be some degree of publication bias (Appendix H in Supplementary Material).

We implemented sensitivity analysis by omitting a single study step by step, and the result did not change, indicating that our result was stable (Appendices I, J in Supplementary Material).

### Methodological Quality of Included Reviews

According to the recommendations of AMSTAR-2, users need to adopt an evaluation process based on key domain identification, namely parts 2, 4, 7, 9, 11, 13, and 15. The results of the AMSTAR-2 evaluation are shown in Table 2. According to the AMSTAR-2 classification, 33 reviews (94%) were of critically low quality, and only one (Cochrane) review was of moderate quality. The essential factors affecting the literature quality included item 2 [only two studies (Peng et al., 2007; Li et al., 2020) “clearly stated that the review methods were established before the conduct of the review and justified any major differences from the protocol”], item 3 [only two studies (Mai and Zheng, 2015; Kwon et al., 2018) “explained their selection of the study designs for inclusion in the review”], item 7 (just 1 study “provided a list of excluded studies and justified the exclusions”), and item 10 (none of the studies “reported on the sources of funding for the studies included in the review”).

**Table 2:** Critical appraisal of included studies using the AMSTAR-2 tool.

**References**	**Q1**	**Q2**	**Q3**	**Q4**	**Q5**	**Q6**	**Q7**	**Q8**	**Q9**	**Q10**	**Q11**	**Q12**	**Q13**	**Q14**	**Q15**	**Q16**	**Overall** ** quality**
Peng et al. (2007)	1	1	0	1	1	1	1	0	1	0	1	1	1	1	1	1	M
Guo et al. (2008)	1	0	0	1	1	1	0	1	1	0	1	1	1	1	1	1	CL
Lee et al. (2008)	1	0	0.5	1	1	1	0	1	1	0	1	1	0	1	0	1	CL
Zhu and Zhang (2009)	1	0	0	1	1	1	0	0	1	0	1	1	1	1	1	1	CL
Yuan and Zhang (2010)	1	0	0	0.5	1	1	0	1	1	0	1	1	1	1	1	0	CL
Liu et al. (2011)	1	0	0	1	0	0	0	1	1	0	1	1	1	1	1	0	CL
Tian et al. (2012)	1	0	0	1	1	1	0	1	1	0	1	1	1	0	1	1	CL
Cao et al. (2013)	1	0	0.5	1	1	1	0	1	1	0	1	1	1	1	1	1	CL
Fang et al. (2014)	1	0	0	1	1	1	0	1	1	0	1	1	1	1	1	1	CL
Zhao et al. (2014)	1	0	0	1	1	1	0	1	1	0	1	1	1	1	1	0	CL
Cao et al. (2014)	1	0	0	0.5	1	1	0	1	1	0	1	1	1	0	0	0	CL
Hu et al. (2014)	1	0	0	1	1	1	0	1	1	0	1	1	1	1	1	0	CL
Zhou et al. (2015)	1	0	0	1	1	1	0	1	1	0	1	1	1	1	1	1	CL
Liu et al. (2015)	1	0	0	0.5	1	1	0	1	1	0	1	0	0	1	0	1	CL
Mai and Zheng (2015)	1	0	1	0.5	0	0	0	1	1	0	1	0	0	1	1	0	CL
Lu et al. (2015)	1	0	0	1	1	1	0	1	1	0	1	1	1	1	1	1	CL
Zhang et al. (2015)	1	0	0	1	1	1	0	1	1	0	1	1	1	1	1	1	CL
Xu and Xie (2015)	1	0	0	1	1	1	0	1	1	0	1	1	1	1	1	1	CL
Deng and Wang (2016)	1	0	0	1	1	1	0	1	1	0	1	1	1	1	1	1	CL
Xiong et al. (2016)	1	0	0	1	1	1	0	1	1	0	1	1	1	1	1	1	CL
Lin et al. (2016)	1	0	0	1	1	1	0	1	1	0	1	1	1	0	0	1	CL
Zou et al. (2016)	1	0	0	1	1	1	0	1	1	0	1	1	0	1	0	0	CL
Zhou et al. (2017)	1	0	0	1	1	1	0	1	1	0	1	1	1	1	1	1	CL
Wan et al. (2017)	1	0	0.5	0.5	1	1	0	1	1	0	1	1	1	1	1	1	CL
Zhan et al. (2017)	1	0	0.5	1	1	1	0	1	1	0	1	1	1	1	1	1	CL
Lin et al. (2017)	1	0	0	1	1	1	0	1	1	0	1	1	0	0	0	1	CL
Kwon et al. (2018)	1	0	1	1	1	1	0	1	1	0	1	1	1	1	1	1	CL
Wu et al. (2018)	1	0	0	0.5	1	1	0	1	1	0	1	1	1	1	1	1	CL
Liu et al. (2018)	1	0	0	1	1	1	0	1	1	0	1	1	1	1	1	1	CL
Li et al. (2018)	1	0	0	1	1	1	0	1	1	0	1	1	1	1	1	0	CL
Kim et al. (2019)	1	0	0	1	1	1	0	1	1	0	1	1	1	1	1	1	CL
Wang et al. (2019)	1	0	0	1	1	1	0	1	1	0	1	1	1	1	1	1	CL
Li et al. (2020)	1	1	0	1	1	1	0	1	1	0	1	1	1	1	1	1	L
Wang et al. (2020)	1	0	0	1	1	1	0	1	1	0	1	1	1	1	1	1	CL
Tang et al. (2020)	1	0	0	1	1	1	0	1	1	0	1	1	1	1	1	1	CL
Number of 1(%)	35 (100)	2 (6)	2 (6)	29 (83)	33 (94)	33 (94)	1 (3)	33 (94)	35 (100)	0(0)	35 (100)	33 (94)	30 (86)	31 (86)	29 (83)	27 (77)	

*1, Yes; 0.5, partial Yes; 0, No; M, Moderate; CL, Critically low; Q1–Q16, See Appendix E in Supplementary Material*.

### Quality of Evidence in Included Reviews (Grading of Recommendations Assessment, Development, and Evaluation)

The 35 SRs/meta-analyses contained 73 outcomes concerned with the effectiveness of acupuncture for MCI or dementia. The results of the Grading of Recommendations Assessment, Development, and Evaluation revealed that 23 outcomes (32%) were of moderate quality, 41 (56%) were low quality, and nine (12%) were very low quality (Table 3). Risk of bias (*n* = 73, 100%) was the most common of the downgrading factors in the included studies, followed by inconsistency (*n* = 35, 48%) and publication bias (*n* = 25, 34%). There were no cases of downgrading due to imprecision or indirectness. This means that the experimental design of the most included studies had potential bias due to randomization, allocation concealment, or blinding methodologies.

**Table 3:** Quality of evidence in included systematic reviews and meta-analyses with Grading of Recommendations Assessment, Development, and Evaluation.

**References**	**Outcomes**		**Limitations**	**Inconsistency**	**Indirectness**	**Imprecision**	**Publication bias**	**Quality of evidence**
Guo et al. (2008)	EA vs. Nimodipine	General effective	Serious^a^	Not serious	Not serious	Not serious	Not serious	Moderate
Lee et al. (2008)	EA vs. Nimodipine/Huperzine/ Perphenazine	MMSE	Serious^a^	Not serious	Not serious	Not serious	Serious^c^	Low
	EA vs. Nimodipine/Huperzine/ Perphenazine	ADL	Serious^a^	Not serious	Not serious	Not serious	Serious^c^	Low
Zhu and Zhang (2009)	EA vs. Nimodipine/Duxil	General effective	Serious^a^	Serious^b^	Not serious	Not serious	Not serious	Low
Yuan and Zhang (2010)	Before and after acupuncture	MMSE	Serious^a^	Not serious	Not serious	Not serious	Not serious	Moderate
Liu et al. (2011)	Acupuncture + Nimodipine/Donepezil/Duxil vs. Nimodipine/Donepezil/Duxil	General effective	Serious^a^	Not serious	Not serious	Not serious	Not serious	Moderate
Cao et al. (2013)	Acupuncture + CFT vs. WM/CFT	MMSE	Serious^a^	Not serious	Not serious	Not serious	Not serious	Moderate
Fang et al. (2014)	Acupuncture + CFT vs. CFT	MMSE	Serious^a^	Not serious	Not serious	Not serious	Not serious	Moderate
Zhao et al. (2014)	EA/ Acupuncture/SA + CFT/ Nimodipine vs. CFT	General effective	Serious^a^	Not serious	Not serious	Not serious	Not serious	Moderate
	Acupuncture vs. CFT	MMSE	Serious^a^	Serious^b^	Not serious	Not serious	Not serious	Low
	Acupuncture vs. CFT	ADL	Serious^a^	Not serious	Not serious	Not serious	Serious^c^	Low
Cao et al. (2014)	Acupuncture vs. Nimodipine/Donepezil/Duxil	MMSE	Serious^a^	Serious^b^	Not serious	Not serious	Serious^c^	Very low
	Acupuncture vs. Nimodipine/Donepezil/Duxil	ADL	Serious^a^	Serious^b^	Not serious	Not serious	Serious^c^	Very low
Hu et al. (2014)	Acupuncture vs. Nimodipine	General effective	Serious^a^	Not serious	Not serious	Not serious	Not serious	Moderate
	Acupuncture vs. Nimodipine	MMSE	Serious^a^	Not serious	Not serious	Not serious	Not serious	Moderate
Zhou et al. (2015)	Acupuncture vs. Nimodipine	MMSE	Serious^a^	Not serious	Not serious	Not serious	Not serious	Moderate
	Acupuncture vs. Nimodipine	ADL	Serious^a^	Not serious	Not serious	Not serious	Not serious	Moderate
Mai and Zheng (2015)	EA vs. CFT	General effective	Serious^a^	Not serious	Not serious	Not serious	Serious^c^	Low
	EA vs. CFT	MMSE	Serious^a^	Not serious	Not serious	Not serious	Serious^c^	Low
	EA vs. CFT	MoCA	Serious^a^	Not serious	Not serious	Not serious	Serious^c^	Low
Lu et al. (2015)	Acupuncture + CT vs. CT	General effective	Serious^a^	Serious^b^	Not serious	Not serious	Not serious	Low
	Acupuncture + CT vs. CT	MMSE	Serious^a^	Not serious	Not serious	Not serious	Serious^c^	Low
	Acupuncture + CT vs. CT	ADL	Serious^a^	Serious^b^	Not serious	Not serious	Not serious	Low
Zhang et al. (2015)	Acupuncture + CFT vs. CFT	General effective	Serious^a^	Serious^b^	Not serious	Not serious	Serious^c^	Very low
	Acupuncture + CFT vs. CFT	MMSE	Serious^a^	Not serious	Not serious	Not serious	Not serious	Moderate
	Acupuncture + CFT vs. CFT	ADL	Serious^a^	Not serious	Not serious	Not serious	Not serious	Moderate
Xu and Xie (2015)	BA + Nimodipine vs. Nimodipine	General effective	Serious^a^	Not serious	Not serious	Not serious	Not serious	Moderate
	BA + Nimodipine vs. Nimodipine	MMSE	Serious^a^	Serious^b^	Not serious	Not serious	Not serious	Low
Deng and Wang (2016)	SEA vs. Nimodipine	General effective	Serious^a^	Not serious	Not serious	Not serious	Serious^c^	Low
	SEA vs. Nimodipine	MMSE	Serious^a^	Not serious	Not serious	Not serious	Serious^c^	Low
Xiong et al. (2016)	SA + CT vs. CT	MMSE	Serious^a^	Serious^b^	Not serious	Not serious	Not serious	Low
Lin et al. (2016)	Acupuncture vs. CT	MMSE	Serious^a^	Serious^b^	Not serious	Not serious	Not serious	Low
	Acupuncture vs. CT	ADL	Serious^a^	Not serious	Not serious	Not serious	Not serious	Moderate
	Acupuncture vs. CT	MoCA	Serious^a^	Not serious	Not serious	Not serious	Serious^c^	Low
Zou et al. (2016)	Acupuncture vs. Huperzine/Nimodipine	General effective	Serious^a^	Not serious	Not serious	Not serious	Not serious	Moderate
	Acupuncture vs. Huperzine/Nimodipine	MMSE	Serious^a^	Serious^b^	Not serious	Not serious	Not serious	Low
	Acupuncture vs. Huperzine/Nimodipine	ADL	Serious^a^	Serious^b^	Not serious	Not serious	Not serious	Low
	Acupuncture vs. Huperzine/Nimodipine	HDS	Serious^a^	Serious^b^	Not serious	Not serious	Not serious	Low
Zhou et al. (2017)	Acupuncture + HM vs. Huperzine/Hydergine/Donepezil	General effective	Serious^a^	Not serious	Not serious	Not serious	Not serious	Moderate
	Acupuncture + HM vs. Huperzine/Hydergine/Donepezil	MMSE	Serious^a^	Not serious	Not serious	Not serious	Not serious	Moderate
	Acupuncture + HM vs. Huperzine/Hydergine/Donepezil	ADL	Serious^a^	Serious^b^	Not serious	Not serious	Serious^c^	Very low
Wan et al. (2017)	EA vs. Nimodipine	General effective	Serious^a^	Serious^b^	Not serious	Not serious	Serious^c^	Very low
	EA vs. Nimodipine	MMSE	Serious^a^	Serious^b^	Not serious	Not serious	Not serious	Low
	EA vs. Nimodipine	ADL	Serious^a^	Not serious	Not serious	Not serious	Serious^c^	Low
Zhan et al. (2017)	EA + Nimodipine/CFT vs. Nimodipine	General effective	Serious^a^	Serious^b^	Not serious	Not serious	Serious^c^	Very low
	EA + Nimodipine/CFT vs. Nimodipine CFT	MMSE	Serious^a^	Serious^b^	Not serious	Not serious	Not serious	Low
	EA + Nimodipine/CFT vs. Nimodipine/CFT	MoCA	Serious^a^	Serious^b^	Not serious	Not serious	Not serious	Low
Lin et al. (2017)	Acupuncture vs. TCM	General effective	Serious^a^	Serious^b^	Not serious	Not serious	Not serious	Low
	Acupuncture vs. TCM	MMSE	Serious^a^	Serious^b^	Not serious	Not serious	Not serious	Low
Kwon et al. (2018)	AA vs. Nimodipine/Almitrine-Raubasine	MMSE	Serious^a^	Serious^b^	Not serious	Not serious	Serious^c^	Very low
	AA vs. Nimodipine/Almitrine-Raubasine	ADL	Serious^a^	Not serious	Not serious	Not serious	Serious^c^	Low
	AA + HM vs. HM	MMSE	Serious^a^	Serious^b^	Not serious	Not serious	Serious^c^	Very low
Wu et al. (2018)	EA vs. Nimodipine	MMSE	Serious^a^	Serious^b^	Not serious	Not serious	Not serious	Low
	EA vs. Nimodipine	ADL	Serious^a^	Serious^b^	Not serious	Not serious	Not serious	Low
Liu et al. (2018)	EA + CT + CFT vs. CT+CFT	MMSE	Serious^a^	Serious^b^	Not serious	Not serious	Not serious	Low
Li et al. (2018)	Acupuncture + Nimodipine vs. Nimodipine	General effective	Serious^a^	Not serious	Not serious	Not serious	Serious^c^	Low
	Acupuncture + Nimodipine vs. Nimodipine	MMSE	Serious^a^	Serious^b^	Not serious	Not serious	Not serious	Low
	Acupuncture + Nimodipine vs. Nimodipine	ADL	Serious^a^	Serious^b^	Not serious	Not serious	Serious^c^	Very low
	Acupuncture + Nimodipine vs. Nimodipine	MoCA	Serious^a^	Serious^b^	Not serious	Not serious	Not serious	Low
Kim et al. (2019)	EA vs. Nimodipine/Donepezil	MMSE	Serious^a^	Not serious	Not serious	Not serious	Not serious	Moderate
	EA vs. Nimodipine/Donepezil	MoCA	Serious^a^	Not serious	Not serious	Not serious	Serious^c^	Low
Wang et al. (2019)	EA vs. Donepezil/Huperzine	MMSE	Serious^a^	Serious^b^	Not serious	Not serious	Not serious	Low
	EA vs. Donepezil/Huperzine	ADL	Serious^a^	Serious^b^	Not serious	Not serious	Not serious	Low
Li et al. (2020)	Acupuncture + Nimodipine/Donepezil vs. Nimodipine/Donepezil	General effective	Serious^a^	Not serious	Not serious	Not serious	Not serious	Moderate
	Acupuncture + Nimodipine/Donepezil vs. Nimodipine/Donepezil	MMSE	Serious^a^	Serious^b^	Not serious	Not serious	Not serious	Low
	Acupuncture + Nimodipine/Donepezil vs. Nimodipine/Donepezil	MoCA	Serious^a^	Serious^b^	Not serious	Not serious	Not serious	Low
	Acupuncture + Nimodipine/Donepezil vs. Nimodipine/Donepezil	ADL	Serious^a^	Serious^b^	Not serious	Not serious	Serious^c^	Low
Wang et al. (2020)	Acupuncture/EA vs. Nimodipine/Donepezil/Huperzine	MMSE	Serious^a^	Serious^b^	Not serious	Not serious	Not serious	Low
	Acupuncture/EA vs. Nimodipine/Donepezil/Huperzine	ADL	Serious^a^	Not serious	Not serious	Not serious	Not serious	Moderate
	Acupuncture/EA vs. Nimodipine/Donepezil/Huperzine	HDS	Serious^a^	Not serious	Not serious	Not serious	Not serious	Moderate
Tang et al. (2020)	Acupuncture vs. CFT (on post-operative day 7)	Incidence of POCI	Serious^a^	Not serious	Not serious	Not serious	Not serious	Moderate
	Acupuncture vs. CFT (on post-operative day 7)	MMSE	Serious^a^	Not serious	Not serious	Not serious	Not serious	Moderate

a *The design of the experiment with a large bias in random, distributive hiding or blind; ^b^The confidence interval overlaps less, the heterogeneity test P is very small, and the I^2^ is larger; ^c^Fewer studies are included, and there may be greater publication bias*.

## Discussion

As far as we know, this is the first SR that has systematically reanalyzed the efficacy of acupuncture for MCI and dementia. Due to the negative and positive results in the included SRs/meta-analyses, we summarized the original RCTs for a new meta-analysis to draw a more systematic conclusion. Moreover, we assessed the quality of the methodological and the quality of the evidence included in the SRs/meta-analyses to afford an evidence-based evaluation and a scientific summary about the effectiveness of acupuncture for MCI and dementia. A total of 35 SRs/meta-analyses were included in this SR, involving 331 original research studies and 22,743 participants (mean age: 62.55 ± 6.22 years), to summarize and analyze the effects of acupuncture on MCI or dementia.

The pooled results of 18 included SRs/meta-analyses (120 RCTs) showed that acupuncture (including EA, scalp acupuncture, body acupuncture, and scalp EA) had a superior effective rate to Western drugs. Our reanalysis of 39 RCTs (*n* = 2,868 participants) reached the same conclusion. MMSE is a sensitive marker for the diagnosis of MCI and dementia (Folstein et al., 1975). The combined results of 31 included SRs/meta-analyses (*n* = 214 RCTs) indicated that acupuncture markedly improved the MMSE score compared with Western drugs, and our reanalysis of 37 RCTs (*n* = 2,271 participants) found that there was a statistically significant difference. In summary, the current proof shows that acupuncture may be more valid than western medicine.

In addition to impaired cognitive function, there are other ways to reflect the symptoms of MCI and dementia. Six studies reported that acupuncture did not improve ADLs (Cao et al., 2014; Zou et al., 2016; Wan et al., 2017; Zhou et al., 2017; Kwon et al., 2018; Wu et al., 2018), and the results of our meta-analysis of 20 RCTs (*n* = 1,248 participants) revealed that, compared with the control group, the ADL score in the experimental group was not significantly improved. However, this result may have been due to the different types of western medicine used and differences in baseline ADL scores (Kwon et al., 2018).

It should be highlighted that the methodological quality of SRs/meta-analyses regarding acupuncture for MCI and dementia is mediocre. The evaluation results of AMSTAR-2 revealed that the majority of the included SRs/meta-analyses were of critically low quality. Few of them clearly stated that the review methods were established before the review, and only one of their authors showed a list of excluded studies and reported on the funding sources for the studies included in the review. These methodological flaws may increase the risk of bias in the review and even affect the final results. Accordingly, researchers should pay attention to compliance with relevant requirements in future studies to increase rigor.

Based on the Grading of Recommendations Assessment, Development, and Evaluation system result, it showed that most outcomes were low or very low quality, and all of them were shown because of the restrictions due to potential bias in randomization, allocation concealment, or blinding. Regarding the latter, it must be acknowledged that double-blinding of acupuncture trials is generally not feasible, and sham controls are relevant only for efficacy studies and not those testing effectiveness relative to no treatment or to another control intervention that is easily distinguishable from acupuncture. Irrespective, this review showed that the greater heterogeneity among articles caused by the small sample size and a few number of included reviews led to a greater publication bias. Thus, further high-quality research with larger sample size is needed in the future to corroborate our conclusions.

Some previous studies have reached the same conclusion. Huang et al. (2020) reviewed the meta-analysis of acupuncture in the treatment of Alzheimer's disease. The review included 11 meta-analyses, and the authors found that acupuncture is a promising complementary therapy, but the methodological evaluation results demonstrated that the included literature quality was low. In the overview by Hou et al. (2020), it was also pointed out that the methodologies and reports of acupuncture treatment of MCI were substandard, and the quality of evidence was poor. Based on the conclusions discussed earlier, RCTs should solve methodological matters via strict experimental designs, reasonable evaluation, and rigorous analyses in the future.

## Strengths and Limitations

We extracted all the original data from the included SRs/meta-analyses and conducted a reanalysis to ensure a large enough sample size and draw the most reliable conclusion possible. However, there are several restrictions to this SR that should be focused. Firstly, the methodological quality assessment and the strength of the evidence are ultimately a subjective process, and the conclusions of the assessment may be subject to the author's subconscious bias. Secondly, we regret that the authors were unable to retrieve any high-quality reviews this time, which is not conducive to enhancing the reliability of the research results.

## Future Perspective

Alone or as an adjunct to other interventions, acupuncture has been effectually used in the clinical practice of treating patients with MCI or dementia. To improve the reliability of study conclusions and direct clinical practice, future study should focus on the following points: (1) A complete research framework should be designed and registered to avoid any risk of bias; (2) the research design should be strictly executed in accordance with standards, such as SR/MA research should strictly abide by the Preferred Reporting Items for Systematic Reviews and Meta-Analyses guidelines; (3) the author should make a clear definition of the type and severity of the diseases involved in the research; (4) in the process of article screening, each step should be recorded in detail, and a list of excluded studies and reasons for exclusion should be listed; (5) funding information should be retained in the review to improve the credibility of the study results; (6) the current research is mainly concentrated in China, and some high-quality reviews on this theme should be performed in other different countries in the future.

## Conclusion

This review was evaluated and reanalyzed according to the principles and methods of evidence-based medicine. Existing evidence suggests that acupuncture is effective for treating MCI and dementia; however, the current SRs/meta-analyses are greatly limited by low methodological quality. Therefore, researchers should improve the scientific research design of future acupuncture RCTs for MCI and dementia to improve their quality and better guide clinical treatment.

## Data Availability Statement

The original contributions presented in the study are included in the article/Supplementary Material, further inquiries can be directed to the corresponding author.

## Author Contributions

WH and WZ planned and designed the study. WH, ML, and XH screened potential researches and extracted data from the included researches. WH and XH appraised the quality and summarized the evidence. WH wrote the manuscript draft. WZ revised the draft. All authors approved the final version of the manuscript.

## Conflict of Interest

The authors declare that the research was conducted in the absence of any commercial or financial relationships that could be construed as a potential conflict of interest.
