# Supplementary material for: Acupuncture for Mild Cognitive Impairment and Dementia: An Overview of Systematic Reviews
Source: Front Aging Neurosci. 2021 May 14;13:647629. doi: 10.3389/fnagi.2021.647629 (PMC8160113; doi:10.3389/fnagi.2021.647629)
Supplement: Supplementary file 1 [file Table_1.DOCX]

Supplementary Material

# Supplementary Figures and Tables

## Supplementary Tables

**Appendix A** Search strategies of PubMed database

| Strategies |  |
| --- | --- |
| #1 | Acupuncture [Mesh] |
| #2 | Acupuncture [ti, ab] OR acupuncture therapy [ti, ab] OR electro-acupuncture [ti, ab] OR acupuncture ear [ti, ab] OR auricular needle [ti, ab] OR auricular acupuncture [ti, ab] OR abdominal acupuncture [ti, ab] OR scalp acupuncture [ti, ab] OR acupuncture points [ti, ab] OR catgut implantation [ti, ab] |
| #3 | (#1 OR #2) |
| #4 | Cognitive Dysfunction [Mesh] |
| #5 | Cognitive Dysfunction [ti, ab] OR Cognitive Impairment [ti, ab] OR Age-Related Memory Disorders [ti, ab] OR Mild Cognitive Impairment [ti, ab] OR MCI [ti, ab] OR Mild Neurocognitive Disorder [ti, ab] OR Cognitive Decline [ti, ab] OR Mental Deterioration [ti, ab] |
| #6 | (#4 OR #5) |
| #7 | Dementia [Mesh] |
| #8 | Dementia [ti, ab] OR Senile Paranoid Dementia [ti, ab] OR Amentia [ti, ab] |
| #9 | (#7 OR #8) |
| #10 | Alzheimer Disease [Mesh] |
| #11 | Alzheimer Disease [ti, ab] OR Alzheimer's Disease [ti, ab] OR Senile Dementia [ti, ab] OR Alzheimer Type Dementia [ti, ab] OR ATD [ti, ab] OR Alzheimer Type Senile Dementia [ti, ab] OR Primary Senile Degenerative Dementia [ti, ab] OR Alzheimer Syndrome [ti, ab] OR Alzheimer Dementia [ti, ab] |
| #12 | (#10 OR #11) |
| #13 | Meta-analysis[Mesh] |
| #14 | Systematic [ti, ab] OR Review [ti, ab] OR Meta-analysis [ti, ab] |
| #15 | (#13 OR #14) |
| #16 | (#6 OR #9 OR #12) |
| #17 | (#3 AND #15 AND #16) |

**Appendix B** Full text articles excluded with reasons

| Full text articles excluded | Reasons |
| --- | --- |
| Liu JL (2009) | Non-systematic review |
| Claire Hulme (2010) | The intervention does not meet the criteria |
| Deren Wang (2011) | Non-systematic review |
| Peng C (2013) | Non-journal article |
| Zhou L (2013) | Data duplication |
| Cui Y (2014) | Not providing sufficient information |
| Xie YY (2014) | The control group not meet the criteria |
| Guoxin Zhang (2015) | The intervention does not meet the criteria |
| Jin YH (2015) | The intervention does not meet the criteria |
| Aiying A. Xiao (2015) | Summary of the meeting |
| Xin M (2015) | Non-journal article |
| Liu F (2015) | Data duplication |
| Li JP (2016) | Non-journal article |
| Chen YB (2016) | Non-journal article |
| Wang Y (2017) | Non-journal article |
| Yang Ye (2017) | Protocol |
| Tatiana-Danai Dimitriou (2017) | The intervention does not meet the criteria |
| Mason Chin Pang Leung (2018) | Non-RCT |
| Fang Liu (2018) | The intervention does not meet the criteria |
| Kim Y (2018) | Summary of the meeting |
| Weipeng Sun (2018) | Protocol |
| Cheng-Hwang Perng (2018) | Non-RCT |
| Soo Ah Sohn (2018) | Non-systematic review |
| Sun KL (2018) | Non-journal article |
| Eun-ji Kim (2018) | Non-Chinese/English |
| Lin FC (2018) | Non-journal article |
| Shujun Shao (2019) | Protocol |
| Cheng-Hao Tu (2019) | Non-systematic review |
| Xin Lai (2020) | The intervention does not meet the criteria |
| Shuqi Yao (2020) | The intervention does not meet the criteria |

**Appendix C** Characteristics of original RCTs

| Included  studies | Participants, Age | Participants, male/female | | Intervention | | Disease types | Acupoints | | Outcome  measures |
| --- | --- | --- | --- | --- | --- | --- | --- | --- | --- |
|  |  | T | C | T | C |  |  |  |  |
| Lai XS 1997 | 63.70±5.40 | 30(22/8) | 30(25/5) | EA, 1 time/daily, 10 days/course | WM, Aniracetam 20mg, 3 times daily | VD | Baihui, Shuigou | | Efficiency |
| Ou YX 1999 | 65.53±6.80 | 16(10/6) | 14(9/5) | AT, 30 min/daily, 6 days/week | WM, Nimodipine 20mg, 20~40mg/time, 3 times/daily | AD | Baihui, Sishencong, Shenyu | | WMS; HDS; ADL |
| Gao HY 1999 | 65.30±5.15 | 31(21/10) | 32(23/9) | AT, 15 min/ daily, 5 days/week | WM, 80mg, 3 times/daily | VD | Sishencong, Renzhong, Renzhong | | Efficiency; HDS |
| Mo FZ 2000 | 65.38±5.76 | 30(19/11) | 30(17/13) | EA, 30 min/daily, 5 days/week | WM, 1.5 mg, 3 times/daily, 5 days/week | VD | Sishencong, Fengchi, Neiguan, Benshen | | Efficiency; HDS; ADL |
| Zhao JX 2000 | 61.00±8.20 | 36(20/16) | 32(17/15) | EA, 20 min/daily, 30 days/course | WM, Hydergine 2mg, 3 times/daily | VD | Baihui, Shenyu | | Efficiency; MMSE; |
| Li ZR 2002 | 65.00±6.00 | 37(22/15) | 14(9/5) | AT, 30 min/daily, 6 days/week | WM, Nimodipine 20mg, 20~40mg/time, 3 times/daily | AD | Baihui, Sishencong, Fengchi, Shenyu | | Efficiency; HDS; MMSE; ADL |
| Dong HT 2002 | - | 11(-) | 10(-) | EA, 40 min/daily, 5 days/week, 4 weeks/course | WM, Huperzine 0.1mg, 2 times/daily, 30days/course | AD | Baihui, Shenyu, Shenmen | | MMSE; ADL |
| Zhou J 2002 | 50~78 | 30(-) | 27(-) | AT, 30 min/time, 10 times/course | WM, Citicoline 1g, 1 time/daily | SAE | Baihui, Fengchi | | Efficiency |
| Yu XX 2003 | - | 34(-) | 34(-) | EA, 30 min/daily, 6 days/week | WM, Nimodipine 30mg, 3 times/daily | VD | Sishencong, Benshen | | Efficiency |
| Wang PQ 2003 | 67.86±4.86 | 30(17/13) | 27(13/17) | AT, 20 min/daily, 5 days/week | WM, Hydergine 2mg, 3 times/daily | VD | Shenyu | | Efficiency |
| Wang DY 2003 | 67.50 | 30(-) | 30(-) | AT, 30 min/daily, 60 days/course | WM, Almitrine and raubasine, 1 time/daily | SAE | Baihui, Guanyuan, Shenmen | | Efficiency; HDS |
| Liu GQ 2003 | 66.20±7.59 | 27(20/7) | 27(19/8) | AT, 1 time/daily, 6 days/week | WM | MID | Baihui, Sishencong, Shenting, Shenyu | | Efficiency; MMSE; ADL |
| Mi JP 2004 | 62.51±5.23 | 32(19/13) | 32(16/16) | AT, 30 min, 10 times/course | WM, Ischolium retard jablets 2.5mg, 2 times/daily | VD | Sishencong | | Efficiency; HDS; ADL |
| Nie ZH 2004 | 67.56±4.78 | 48(28/20) | 48(29/19) | AT, 30 min/daily, 10 times/course | WM, Citicoline 0.75g, 1 time/daily | VD | Baihui, Shenting, Benshen, Renzhong | | Efficiency; HDS |
| Qi HM 2004 | 55~78 | 36(-) | 24(-) | AT, 20~30 min/daily, 10 times/course | WM, 3 times/daily | VD | Yamen, Tianzhu, Taichong | | Efficiency; MMSE; HDS |
| Lun X 2004 | 64.56±7.43 | 25(-) | 25(-) | AT, 30 min/daily, 5 times/week | WM, Nimodipine 30mg, 3 times/daily | MID | Fenglong, Quchi | | Efficiency |
| Xu YX 2005 | - | 25(-) | 20(-) | EA, 30 min/daily, 5 times/week | WM, Nimodipine 30mg, 3 times/daily | DCI | Sishencong, Baihui | | Efficiency |
| Wang M 2005 | 50~75 | 31(20/11) | 30(20/10) | AT, 1 time/daily, 5days/week | WM, Hydroergot, Pyrithiol hydrochloride | VD | Shenting, Benshen, Sishencong, Baihui | | Efficiency; HDS; MMSE; ADL |
| Zhu BP 2005 | 41.00±5.00 | 52(40/12) | 54(41/13) | EA, 20 min/daily | WM, Huperzine 0.1mg | VD | Yamen, Jiaosun, Fengchi, Touwei | | Efficiency; MMSE |
| Luo DH 2006 | 67.70±7.20 | 48(-) | 48(-) | EA, 25 min, 25 days/course | WM, Hydergine 50mg, 3 times/daily, 30 days/course | AD | Shenyu, Zusanli, Taixi, Dazhui | | Efficiency; MMSE |
| Zhang H 2006 | 45~80 | 24(15/9) | 28(17/11) | EA, 30 min/daily, 5 times/week | WM, Nimodipine 20mg, 3 times/daily | VD | Sishencong, Baihui, Shenting, Fengchi | | Efficiency; MMSE; ADL |
| Li SZ 2006 | 65.8±3.20 | 30(18/12) | 28(17/11) | EA, 60 min/daily, 30 days/course | WM, Piracetam 0.4g, 3 times/daily | VD | | Sishencong, Fengchi | Efficiency; MMSE; HDS |
| Ma XP 2006 | 65.6±4.78 | 28(15/13) | 20(12/8) | EA, 30 min/daily, 10 times/course | WM, Citicoline 0.5g, 10 times/course | VD | Sishencong, Yintang | | Efficiency |
| Yu XG 2007 | 63.30 | 25(14/11) | 26(15/11) | AT, 20~25 min, 5 times/week+WM, Nimodipine 30mg, 3 times/daily | WM, Nimodipine 30mg, 3 times/daily | MCI | Baihui, Shenting, Fengfu, Yintang | | Efficiency; MMSE; ADL |
| Sun YZ 2007 | 65.70 | 30(16/14) | 29(14/15) | AT, 30 min, 6 times/week+WM, Donepezil 2.5mg/daily | WM, Donepezil 2.5mg/daily | MCI | Baihui, Guanyuan, Shenmen, Taixi | | Efficiency |
| Zhao LG 2007 | 67.00±2.12 | 16(-) | 16(-) | AT, 40 min | WM, Nimodipine 20mg, 3 times/daily | AD | Baihui, Dazhui | | Efficiency; MMSE; ADL; HDS |
| Liu ZB 2008 | 69.16±2.12 | 40(18/22) | 40(19/21) | AT, 10 min/daily, 5 days/week | WM, Duxil 40mg, 2 times/daily | AD | Yingxiang, Yintang | | Efficiency; MMSE; HDS |
| Huang F 2008 | 61.05±9.68 | 40(21/19) | 40(20/20) | AT, 30 min/daily+CFT | CFT | VD | Baihui, Renzhong, Shenmen | | MMSE |
| He X 2008 | 65.78±6.42 | 30(-) | 30(-) | AT, 1 times/Ddaily+WM | WM | MCI | Neiguan, Sanyinjiao, Baihui, Yintang | | Efficiency; MMSE; HDS |
| Peng XH 2009 | 63.71±9.32 | 27(19/8) | 26(17/9) | EA, 30 min/daily, 4 times/week | WM, Nimodipine 20mg, 3 times/daily | VD | Sishencong, Baihui, Shenting, Fengchi | | MMSE; ADL |
| Sun YZ 2009 | 67.20±4.30 | 30(19/11) | 30(13/17) | AT, 40 min+WM | WM, Duxil 40mg, 2 times/daily | MCI | Baihui, Neiguan, Sanyinjiao | | Efficiency; MMSE |
| Zhu H 2010 | 72.30±6.00 | 20(-) | 20(-) | AT, 30 min/daily, 7 times/week | WM, Donepezil 5 mg/daily | AD | Baihui, Shenyu | | MMSE |
| Wang Y 2010 | 67.63±6.44 | 29(-) | 28 (-) | AT +CT | CT | MCI | — | | Efficiency |
| Li T 2010 | - | 30(-) | 28 (-) | AT, 20 min, 10 days/course+WM | WM | MCI | Sishencong, Fengchi, Neiguan, Baihui | | Efficiency; MMSE; ADL |
| Yin JQ 2011 | 62.67±5.10 | 30(17/13) | 30(18/12) | EA, 30 min/daily, 5 times/week | WM, Nimodipine 20mg, 3 times/daily | VD | Sishencong, Shenting, Baihui | | MMSE |
| Jiao ZH 2011 | 64.30 | 30(-) | 30 (-) | AT, 30 min/daily+WM, Donepezil | WM, Donepezil 5 mg/daily | MCI | Baihui, Sishencong | | MMSE; ADL |
| Zhao H 2011 | 45~70 | 24(16/8) | 24(12/12) | AT, 30 min/daily+WM, Donepezil | WM, Donepezil 5 mg/daily | VD | Baihui, Fengchi | | MMSE; ADL |
| Zhao L 2012 | - | 94(46/48) | 93(44/49) | EA, 30 min/time, 3 times/week | WM, Nimodipine 30mg, 3 times/daily | MCI | Shenting, Baihui, Sishencong, Fengchi | | Efficiency; MMSE |
| Tong X 2012 | 58.40±8.50 | 34(21/13) | 34(24/10) | AT, 40 min, 2 times/daily+CT | CT | MCI | Baihui, Sishencong, Neiguan, Shuigou | | Efficiency; MMSE |
| Liu XJ 2012 | 60.50 | 120(61/59) | 120(62/58) | AT, 30 min/daily, 5 days/week+CT | CT | MCI | Yintang, Shangxing, Neiguan, Renzhong | | Efficiency; ADL |
| Zhao Y 2012 | 67.80 | 30(17/13) | 30(16/14) | AT, 30 min, 3 times/week +WM, Nimodipine | WM, Nimodipine 30mg, 3 times/daily | MCI | Fengchi, Shenting, Sishencong, Baihui | | MMSE |
| Zhao YH 2012 | 56.34±9.57 | 30(16/14) | 30(18/12) | AT, 4 times/week+WM, Nimodipine | WM, Nimodipine 30mg, 3 times/daily | MCI | Sishencong, Fengchi, Benshen, Shenting | | MMSE |
| Li W 2012 | 40~79 | 48(30/18) | 46(24/22) | AT, 30 min, 5 times/week+WM, Nimodipine | WM, Nimodipine 30mg, 3 times/daily | MCI | Baihui, Shenting, Qucha, Shencong | | Efficiency; MMSE |
| Yin HK 2013 | 60~85 | 30(-) | 30(-) | AT, 45 min /week+WM, Aricept | WM, Aricept 5mg/daily | AD | Qianding, Baihui | | MMSE; ADL |
| Lin F 2013 | 66~78 | 34(16/18) | 34(14/20) | AT, 40 min/daily+WM, Duxil | WM, Duxil | MCI | Sishencong, Baihui | | Efficiency |
| Wang Y 2014 | 70.70±9.10 | 28(13/15) | 27(14/13) | AT, 30 min/daily, 10 days/course+WM, Donepezil | WM, Donepezil 5~10 mg/daily, 10 days/course | AD | Yintang | | MMSE |
| Lin CR 2014 | 73.44±3.37 | 18(7/11) | 18(8/10) | EA, 30min/daily, 6 times/week | WM, Aricept 5 mg/daily | AD | Baihui, Sishencong | | MMSE; ADL |
| Li QW 2015 | 65.10±11.30 | 28(16/12) | 28(15/13) | EA, 30min/daily, 5 times/week | WM, Nimodipine 30mg, 3 times/daily | VD | Shenting, Benshen | | MMSE; HDS; ADL |
| Zhu CF 2015 | 62.13±7.99 | 30(17/13) | 30(16/14) | AT, 30 min/daily, 6 days/week | WM, Nimodipine 30mg, 3 times/daily | MCI | Baihui, Fengfu | | MMSE |
| Peng J 2017 | 69.40±5.40 | 25(12/13) | 25(12/13) | AT, 25 min/daily | WM, huperzine 0.2 mg/daily | AD | Shenting, Baihui | | MMSE |
| Jia YJ 2017 | 75.11±6.53 | 35(19/16) | 36(17/18) | AT, 3 times/week+WM, Aricept | WM, Aricept 5 mg/daily | AD | Danzhong, Qihai | | MMSE; ADL |
| Chen DR 2018 | 74.36±5.47 | 48(19/29) | 48(18/30) | AT, 2 times/daily | WM, Donepezil 5 mg/daily | AD | Baihui, Renzhong, Neiguan, Sanyinjiao | | Efficiency; MMSE;ADL |

**Appendix D** The effectiveness of acupuncture on patients with MCI or dementia: systematic review of meta-analysis results

| Author(s) | Studies, n (patients, n) | Comparison | Pooled results (95% CI) | Heterogeneity I^2^ (%) |
| --- | --- | --- | --- | --- |
| **General effective** | | | | |
| Guo XX 2008 | 19(1229) | EA versus WM | OR: 3.72(2.73, 5.07) | 0 |
| Zhu MJ 2009 | 7(517) | EA versus WM | OR: 2.94(1.86, 4.64) | 61.6 |
| Liu JH 2011 | 6(330) | AT+WM versus WM | OR: 3.12(1.96, 4.96) | 0 |
| Zhao D 2014 | 6(395) | EA/AT/SA+CFT/WM versus Non-AT | OR: 4.19(2.58, 6.80) | 0 |
| Hu J 2014 | 9(693) | AT versus WM | OR: 2.89(2.10, 3.97) | 5 |
| Liu WL 2015 | 4(NR) | EA versus CFT | OR: 2.64(1.40, 5.00) | 0 |
| Mai W 2015 | 3(445) | EA versus CFT | RR: 1.53(1.25, 1.89) | 49 |
| Lu JH 2015 | 7(666) | AT+CT versus CT | OR: 6.12(4.01, 9.35) | 64 |
| Zhang Y 2015 | 3(239) | AT+CFT versus CFT | RR: 1.58(1.10, 2.26) | 71 |
| Xu XT 2015 | 7(524) | BA+WM versus WM | RR: 1.25(1.14, 1.38) | 0 |
| Min Deng 2016 | 3(193) | SEA versus WM | OR: 1.78(1.19, 2.65) | 0 |
| Zou JF 2016 | 6(186) | AT versus WM | OR: 1.15(0.69, 1.91) | 0 |
| Simin Zhou 2017 | 12(1028) | AT+HM versus WM | OR: 2.72(2.04, 3.62) | 47 |
| Wan T 2017 | 3(264) | EA versus WM | OR: 4.97(2.89, 8.56) | 52 |
| Zhan J 2017 | 3(191) | EA+WM/CFT versus WM/CFT | RR: 1.37(0.98, 1.91) | 69 |
| Lin L 2017 | 8(613) | AT versus Drug | RR: 1.16(1.03, 1.31) | 56 |
| Li SY 2018 | 2(128) | AT+WM versus WM | OR: 2.38(1.11, 5.11) | 0 |
| Weitong Li 2020 | 12(893) | AT+WM versus WM | OR: 2.52(1.86, 3.42) | 0 |
| **MMSE** | | | | |
| M. S. Lee 2008 | 2(72) | EA versus WM | MD: -0.55(-1.31, 0.21) | 0 |
| Yuan SS 2010 | 4(292) | Before and after AT | OR: 8.59(4.76, 15.51) | 46.5 |
| Huijuan Cao 2013 | 6(340) | AT+CFT versus WM/CFT | MD: 1.99(1.09, 4.31) | 47 |
| Fang Liu 2014 | 4(232) | AT+CFT versus CFT | MD: 3.14(2.06, 4.21) | 36 |
| Zhao D 2014 | 5(364) | AT versus Non-AT | MD: 2.49(0.95, 4.04) | 80 |
| Cao F 2014 | 3(171) | AT versus WM | MD: -0.61(-1.34, 0.13) | 63 |
| Hu J 2014 | 12(927) | AT versus WM | MD: 1.32(0.86, 1.78) | 75 |
| Jing Zhou 2015 | 6(359) | AT versus WM | MD: 1.05(0.16, 1.93) | 57 |
| Liu WL 2015 | 8(NR) | EA versus CFT | MD: 2.12(0.16, 4.08) | 95 |
| Mai W 2015 | 3(445) | EA versus CFT | MD: 1.33(0.85, 1.82) | 75 |
| Lu JH 2015 | 3(200) | AT+CT versus CT | MD: 2.21(1.02, 3.40) | 0 |
| Zhang Y 2015 | 5(383) | AT+CFT versus CFT | MD: 2.64(1.78, 3.50) | 0 |
| Xu XT 2015 | 4(251) | BA+WM versus WM | MD: 2.87(0.64, 5.10) | 87 |
| Min Deng 2016 | 3(394) | Scalp EA versus WM | MD: 1.09(0.55, 1.63) | 78 |
| Xiong J 2016 | 10(732) | SA+CT versus CT | MD: 2.22(1.38, 3.07) | 76 |
| Lin XL 2016 | 19(NR) | AT versus CT | WMD: 4.01(2.32, 5.69) | 95 |
| Zou JF 2016 | 7(319) | AT versus WM | MD: 0.40(-2.18, 2.97) | 100 |
| Simin Zhou 2017 | 11(774) | AT+HM versus WM | MD: 2.10(0.69, 3.51) | 89 |
| Wan T 2017 | 6(435) | EA versus WM | MD: 1.92(0.82, 3.03) | 59 |
| Zhan J 2017 | 10(561) | EA+WM/CFT versus WM/CFT | MD: 1.78(0.24, 3.32) | 93 |
| Lin L 2017 | 5(263) | AT versus Drug | MD: -0.99(-3.45, 1.46) | 77 |
| CY Kwon 2018 | 3(403) | AA versus WM | MD: 0.73(-0.02, 1.48) | 61 |
| CY Kwon 2018 | 2(124) | AA+HM versus HM | MD: 1.31(0.13, 2.49) | 70 |
| Wu JM 2018 | 8(537) | EA versus WM | MD: 1.84(0.85, 2.82) | 55 |
| Liu F 2018 | 15(1000) | EA+CT+CFT versus CT+CFT | MD: 3.37(1.70, 5.05) | 96 |
| Li SY 2018 | 9(606) | AT+WM versus WM | MD: 1.47(1.27, 1.66) | 86 |
| Hwan Kim 2019 | 6(265) | EA versus WM | MD: 0.65(0.28, 1.01) | 0 |
| Wang YS 2019 | 8(472) | EA versus WM | SMD: 0.76(0.42, 1.10) | 68 |
| Weitong Li 2020 | 8(560) | AT+WM versus WM | MD: 1.53(1.04, 2.01) | 63 |
| YunYun Wang 2020 | 14(820) | AT/EA+Drug versus WM | MD: 2.96(1.80, 4.13) | 88 |
| Yidan Tang 2020 | 5(360) | AT versus Non-AT | MD: 2.52(2.18, 2.87) | 28 |
| **ADL** | | | | |
| M. S. Lee 2008 | 2(72) | EA versus WM | MD: -1.29(-1.77, -0.80) | 0 |
| Zhao D 2014 | 3(240) | AT versus Non-AT | MD: 4.53(1.00, 8.06) | 0 |
| Cao F 2014 | 3(113) | AT versus WM | MD: -0.48(-1.72, 0.76) | 86 |
| Jing Zhou 2015 | 4(183) | AT versus WM | MD: -2.80(-4.57, -1.02) | 0 |
| Lu JH 2015 | 6(280) | AT+CT versus CT | MD: 8.78(7.56, 10.00) | 59 |
| Zhang Y 2015 | 5(364) | AT+CFT versus CFT | SMD: 0.52(0.31, 0.73) | 46 |
| Lin XL 2016 | 6(NR) | AT versus CT | WMD: 9.29(5.63, 12.95) | 72 |
| Zou JF 2016 | 5(229) | AT versus WM | MD: 0.60(-0.54, 1.74) | 95 |
| Simin Zhou 2017 | 3(174) | AT+HM versus WM | MD: -3.59(-7.18, 0.01) | 81 |
| Wan T 2017 | 3(264) | EA versus WM | MD: -1.55(-4.65, 1.54) | 33 |
| CY Kwon 2018 | 2(247) | AA versus WM | MD: 0.20(-3.51, 3.91) | 0 |
| Wu JM 2018 | 6(416) | EA versus WM | MD: 0.58(-3.60, 4.76) | 83 |
| Li SY 2018 | 3(218) | AT+WM versus WM | MD: 2.00(0.88, 3.12) | 71 |
| Wang YS 2019 | 3(186) | EA versus WM | SMD: -0.32(-0.61, -0.03) | 0 |
| Weitong Li 2020 | 2(140) | AT+WM versus WM | MD: 1.71(-1.38, 4.79) | 92 |
| YunYun Wang 2020 | 11(497) | AT+WM versus WM (on post-operative day 7) | MD: 0.21(-0.74, 1.16) | 35 |

Note: OR: odds ratio; RR: risk ratio; MD, mean difference; SMD, standard mean difference.

**Appendix E**

| Q1 | Did the research questions and inclusion criteria for the review include the components of PICO? |
| --- | --- |
| Q2 | Did the report of the review contain an explicit statement that the review methods were established prior to the conduct of the review and did the report justify any significant deviations from the protocol? |
| Q3 | Did the review authors explain their selection of the study designs for inclusion in the review? |
| Q4 | Did the review authors use a comprehensive literature search strategy? |
| Q5 | Did the review authors perform study selection in duplicate? |
| Q6 | Did the review authors perform data extraction in duplicate? |
| Q7 | Did the review authors provide a list of excluded studies and justify the exclusions? |
| Q8 | Did the review authors describe the included studies in adequate detail? |
| Q9 | Did the review authors use a satisfactory technique for assessing the risk of bias (RoB) in individual studies that were included in the review? |
| Q10 | Did the review authors report on the sources of funding for the studies included in the review? |
| Q11 | If meta-analysis was performed did the review authors use appropriate methods for statistical combination of results? |
| Q12 | If meta-analysis was performed, did the review authors assess the potential impact of RoB in individual studies on the results of the meta-analysis or other evidence synthesis? |
| Q13 | Did the review authors account for RoB in individual studies when interpreting/discussing the results of the review? |
| Q14 | Did the review authors provide a satisfactory explanation for, and discussion of, any heterogeneity observed in the results of the review? |
| Q15 | If they performed quantitative synthesis did the review authors carry out an adequate investigation of publication bias (small study bias) and discuss its likely impact on the results of the review? |
| Q16 | Did the review authors report any potential sources of conflict of interest, including any funding they received for conducting the review? |

## Supplementary Figures


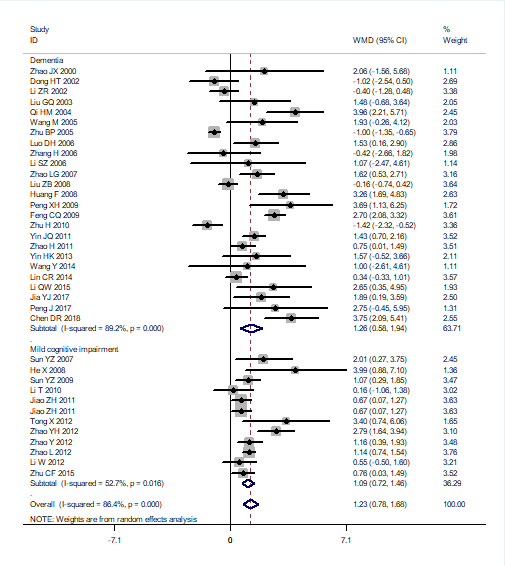


**Appendix F** Forest plot of the effect of acupuncture on the MMSE score using random model


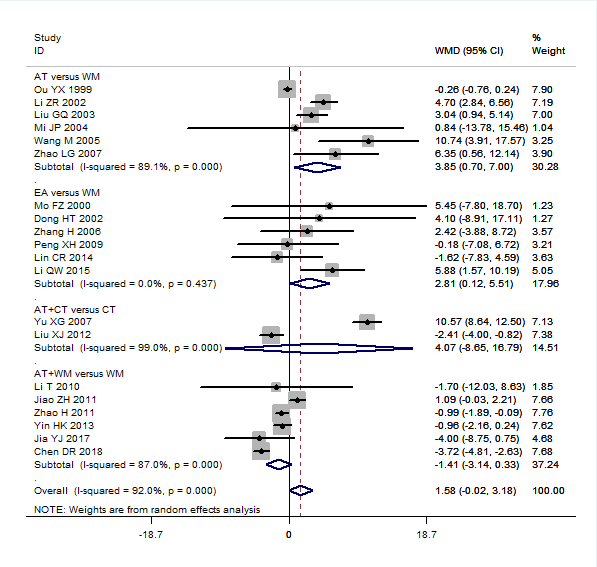


**Appendix G** Forest plot of the effect of acupuncture on the ADL score using random model


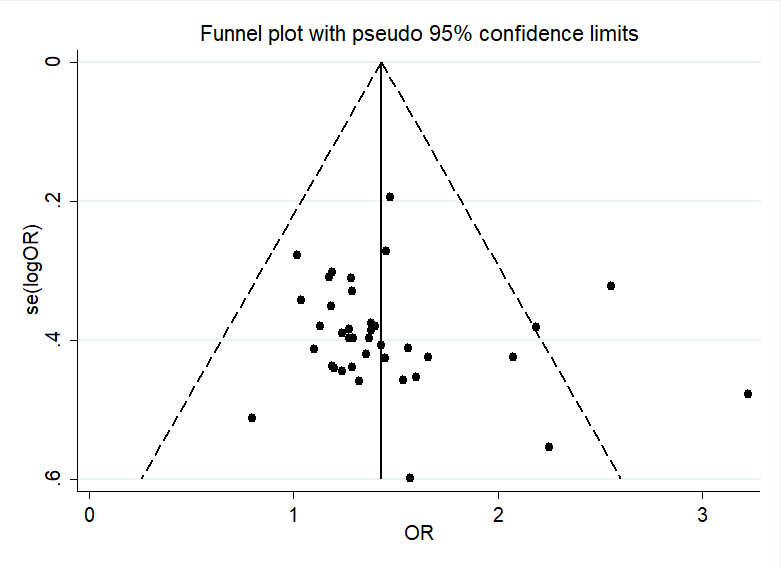


**Appendix H** Funnel plots


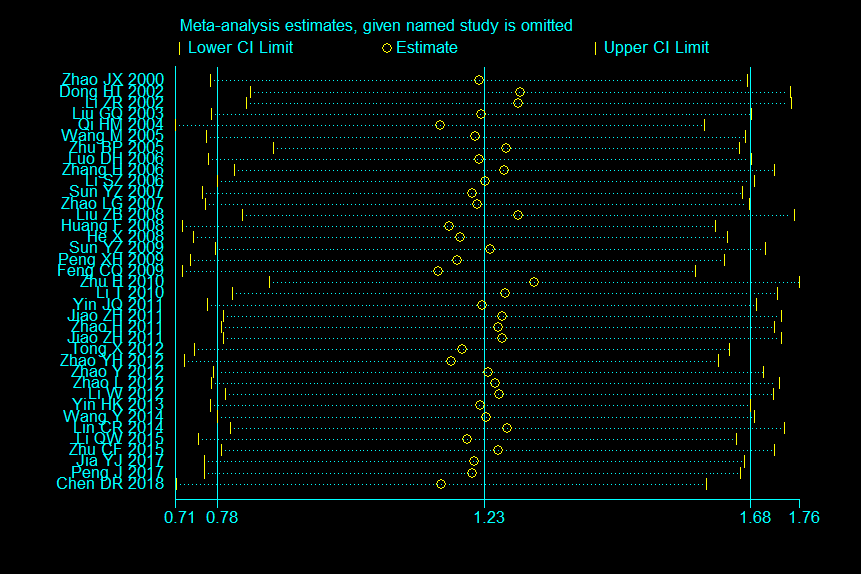


**Appendix I** Sensitivity analysis (MMSE)


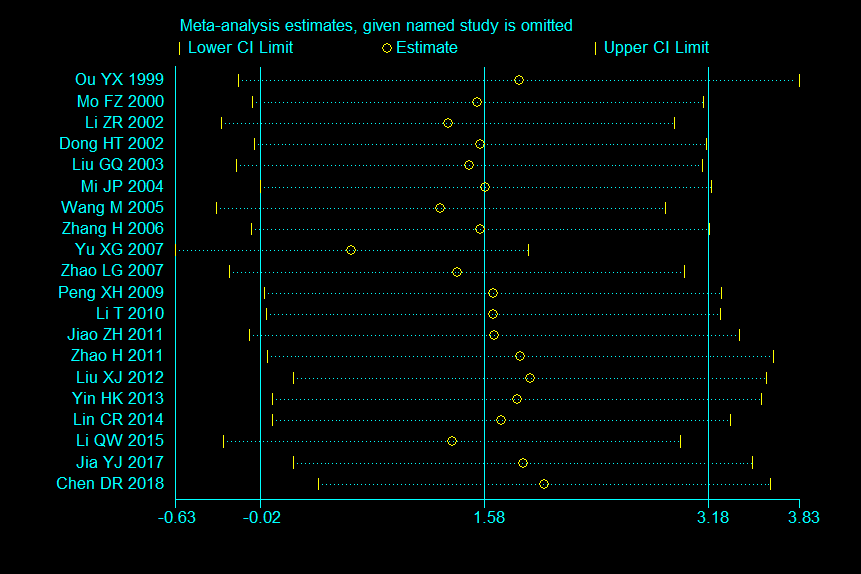


**Appendix J** Sensitivity analysis (ADL)
